# Doughty-electronegative heteroatom-induced defective MoS_2_ for the hydrogen evolution reaction

**DOI:** 10.3389/fchem.2022.1064752

**Published:** 2022-11-23

**Authors:** Zhaohui Xiao, Shengdao Luo, Wei Duan, Xu Zhang, Shixing Han, Yipu Liu, Liang Yang, Shiwei Lin

**Affiliations:** ^1^ State Key Laboratory of Marine Resource Utilization in South China Sea, School of Materials Science and Engineering, Hainan University, Haikou, China; ^2^ State Key Laboratory of Chem/Bio-Sensing and Chemometrics, College of Chemistry and Chemical Engineering, Hunan University, Changsha, China

**Keywords:** defect-induced, molybdenum disulfide, electronegative, electrocatalysts, hydrogen evolution reaction

## Abstract

Producing hydrogen through water electrolysis is one of the most promising green energy storage and conversion technologies for the long-term development of energy-related hydrogen technologies. MoS_2_ is a very promising electrocatalyst which may replace precious metal catalysts for the hydrogen evolution reaction (HER). In this work, doughty-electronegative heteroatom defects (halogen atoms such as chlorine, fluorine, and nitrogen) were successfully introduced in MoS_2_ by using a large-scale, green, and simple ball milling strategy to alter its electronic structure. The physicochemical properties (morphology, crystallization, chemical composition, and electronic structure) of the doughty-electronegative heteroatom-induced defective MoS_2_ (N/Cl-MoS_2_) were identified using SEM, TEM, Raman, XRD, and XPS. Furthermore, compared with bulk pristine MoS_2_, the HER activity of N/Cl-MoS_2_ significantly increased from 442 mV to 280 mV at a current of 10 mA cm^−2^. Ball milling not only effectively reduced the size of the catalyst material, but also exposed more active sites. More importantly, the introduced doughty-electronegative heteroatom optimized the electronic structure of the catalyst. Therefore, the doughty-electronegative heteroatom induced by mechanical ball milling provides a useful reference for the large-scale production of green, efficient, and low-cost catalyst materials.

## 1 Introduction

Hydrogen production from water splitting has become the mainstream process to meet the sustainable development of society, energy, and the environment. (Zou and Zhang, 2015) Various strategies have been developed to produce hydrogen, of which, photo-electrocatalytic and electrocatalytic water splitting were shown to be efficient and clean high-purity hydrogen production technologies. (Benck et al., 2014) Precious metal-based catalysts are the most active electrocatalysts for the hydrogen evolution reaction (HER); however, their large-scale utilization is limited by their relative scarcities and high costs. (Wu et al., 2013) The development of non-noble metal HER catalysts is thus imperative. The two-dimensional material molybdenum disulfide (MoS_2_), a transition metal sulfide, has exhibited a promising HER catalytic activity, similar to that of the precious metal Pt. (Jaramillo et al., 2007; Sun et al., 2022) However, previous research demonstrated that the electrocatalytic activity of pristine MoS_2_ is poor for the HER, due to its inferior electrical conductivity. (Hinnemann et al., 2005; Xu et al., 2016) Increasing the number of catalytically active sites as well as the electrical conductivity of MoS_2_ is expected to improve its intrinsic activity for the HER. (Li et al., 2018) A conventional strategy to realize this is to expose more edge-sulfur atoms in order to increase the number of active sites. For example, previous studies have shown that MoS_2_ materials benefit from more active edge sites when their size is brought down to the nanometer scale. (Chen et al., 2011; Wang et al., 2013) In particular, defect engineering is also an important strategy to modulate the electronic structures of electrocatalysts for enhanced electrocatalytic intrinsic activities. (Xie et al., 2019) For example, defective MoS_2_ was obtained by adjusting the ratio of precursors (S and Mo), with a markedly improve electrocatalytic HER activity achieved. (Xie et al., 2013; Xu et al., 2016) Additionally, strategies were also developed to increase the electronic conductivity of MoS_2_ by introducing composites or heteroatoms. (Guo et al., 2015; Peto et al., 2018) For example, Dai’s group reported the growth of MoS_2_ nanoparticles on reduced graphene oxide, with an impressive catalytic HER activity obtained. (Li et al., 2011) Yang’s group reported that MoS_2_ nanosheets supported by amorphous carbon exhibited an improved catalytic performance for the HER. (Yang et al., 2016) In addition, Wang et al. constructed defective structure-affluent MoS_2_ using oxygen plasma technology to enhance the electronic conductivity and augment the number of active edge sites. (Tao et al., 2015) These previous studies verified that the intrinsic activity of MoS_2_ can be greatly enhanced by intentionally introducing defects. Therefore, inducing defects is indeed an effective strategy to optimize the activities of MoS_2_ materials.

The electronegativity of the doped atoms may play a unique role in the modulation of the electronic structure of the catalyst. (Zheng et al., 2014) However, there are few reports on the different electronegativities of introduced heteroatoms and how they affect the electrocatalyst activity. Halogen atoms, especially chlorine and fluorine, both of which exhibit strong electronegativities, are expected to significantly modulate the electronic structure of catalysts after doping. (Zhang et al., 2021b) In addition, bulk two-dimensional materials can be reduced to nanoscale sizes by the ball milling shear force; physical or chemical doping of the material’s defect structure can be realized during the mechanical ball milling process. (Buzaglo et al., 2017) Previous work demonstrated that edge-selective heteroatom-doped defects can be obtained by ball milling a mixture containing a two-dimensional material and an impurity atom source. (Xiao et al., 2016) For example, the Dai team used ball milling to mix graphite and non-metallic elements, obtaining heteroatom-doped graphene. (Jeon et al., 2013) Molybdenum disulfide and graphite are two-dimensional materials with layered structures. It can be expected that non-metallic target atoms can also be doped into the MoS_2_ crystal structure during the ball milling process.

Herein, we explore the doughty-electronegative heteroatom-induced defect structure of MoS_2_, in which the commercial bulk MoS_2_ incorporated strongly electronegative halogen atoms during mechanical ball milling processing. Ball milling not only reduced the size of the bulk material and made it thinner, but also exposed more active sites for catalytic reaction. At the same time, the doped defect heteroatom regulated and optimized both the surface and the electronic structure of MoS_2_. Thus, its intrinsic activity was significantly increased. Compared with bulk pristine MoS_2_, the HER activity of defect-induced MoS_2_ significantly increased from 442 mV to 280 mV at a current of 10 mA cm^−2^. The defect-induced effect of electronegative heteroatoms through ball milling provides a new design strategy to prepare effective HER catalyst materials, providing a useful reference for the research, development, and commercial mass production of high-performance catalysts.

## 2 Results and discussion

**FIGURE 1:**
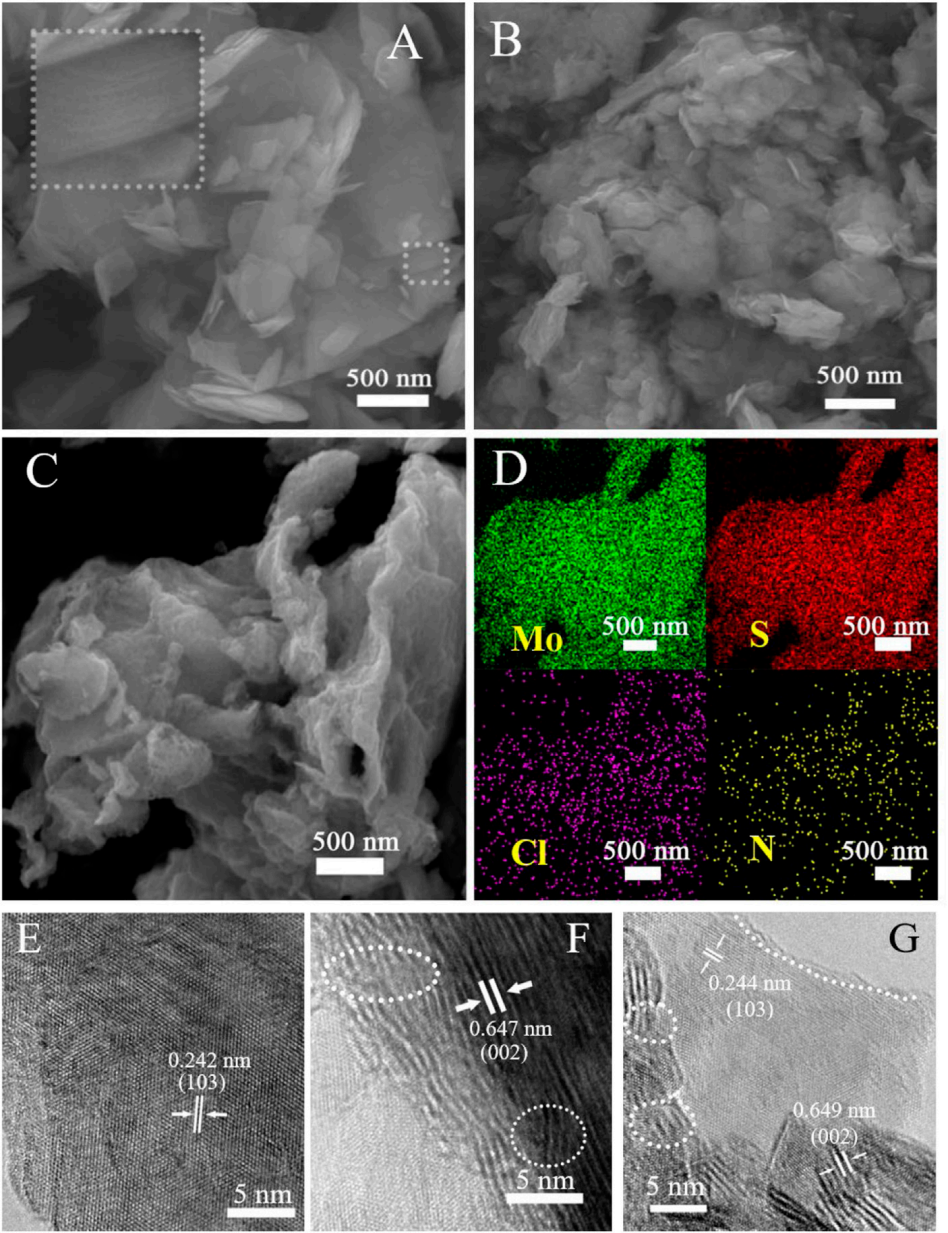
SEM images of **(A)** pristine MoS_2_, **(B)** BM-MoS_2_, and **(C)** N/Cl-MoS_2_. **(D)** EDS elemental mapping of N/Cl-MoS_2_ from **(C)**. HR-TEM images of **(E)** pristine MoS_2_, **(F)** BM-MoS_2_, and **(G)** N/Cl-MoS_2_.

Doughty-electronegative heteroatom-induced defective MoS_2_ materials were prepared by simple mixed ball milling (see the Experimental section in the Supplementary Material S1). Briefly, bulk commercial MoS_2_ (denoted as pristine MoS_2_) and a halogen source containing doughty-electronegative elements (for example, NH_4_Cl as the N and Cl source) were mixed and ball-milled, followed by removal of the residual by-products and impurities. (Xue et al., 2015) The final catalyst product obtained was denoted as N/Cl-MoS_2_. Analogously, non-doping defective MoS_2_ was obtained (denoted as BM-MoS_2_) by directly ball milling bulk commercial MoS_2_ with no halogen source present. The morphologies of the as-prepared MoS_2_ samples were firstly examined by scanning electron microscopic (SEM) and high-resolution transmission electron microscopy (HR-TEM), and the results are shown in Figure 1. Pristine MoS_2_ (Figure 1A) exhibits large-sized nanosheets that were closely packed to on another, resulting in fewer exposed active sites. However, compared with pristine MoS_2_, the BM-MoS_2_ (Figure 1B) and N/Cl-MoS_2_ (Figure 1C) were fragmented to the nanoscale, which was caused by ball milling with or without a halogen source. In strong contrast to pristine MoS_2_ which was on the micron size scale, the sizes of the BM-MoS_2_ and N/Cl-MoS_2_ decreased to a few hundred nanometers and exhibited exfoliated layered structures. SEM-EDS elemental mapping analysis was performed to confirm the contents of N and Cl, and the results are shown in Figure 1D and Supplementary Table S1. The results show that nitrogen and chlorine were successfully doped into the MoS_2_; the amounts of nitrogen and chlorine in the N/Cl-MoS_2_ were 6.04% and 4.00%, respectively. These results are consistent with previous studies on ball milling. (Buzaglo et al., 2017) Furthermore, the progressive morphologies of three catalyst materials were distinguished by HR-TEM imaging. Pristine MoS_2_ exhibits a relatively complete crystal structure, as shown in Figure 1E. An interplanar spacing of 0.242 nm was observed, which was consistent with the d spacing of the (103) planes of hexagonal MoS_2_. (Sun et al., 2022) The interplanar spacing (0.647 nm) was also observed in the HR-TEM image of MoS_2_ with no halogen doping, as shown in Figure 1F. However, the laminar structure was damaged, as indicated by the white dotted lines, indicating that edge faults are obtained by the ball milling shear force. As shown in Figure 1G, after nitrogen and chlorine were doped into the N/Cl-MoS_2_ by ball milling, in addition to the main lattice spacing (0.244 nm) corresponding to the (103) crystal plane structure, a lattice spacing of 0.649 nm corresponding to the (002) crystal plane structure was found to exist. This indicates that in the presence of a halogen precursor, ball milling produced a unique N/Cl-MoS_2_ product with more exposed surface structures, signifying that a variety of defect structures were introduced. After careful observation, the product was found to be relatively disordered in terms of the crystal structure. A reasonable explanation for this is that halogen atoms introduced to the MoS_2_ crystal structure formed localized areas of N-Mo and Cl-Mo bonds during ball milling, in which the original electronic structure was altered, forming unique locally disordered lattice structures. Thus, a novel, defective MoS_2_ structure was prepared, which should display a significantly superior intrinsic HER activity.

**FIGURE 2:**
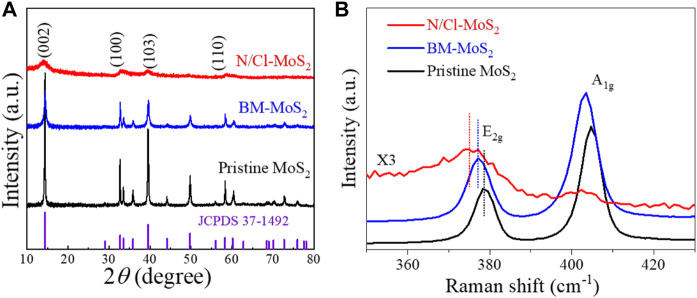
**(A)** XRD patterns of pristine MoS_2_, BM-MoS_2_, and N/Cl-MoS_2_. **(B)** Raman spectra of pristine MoS_2_, BM-MoS_2_, and N/Cl-MoS_2_.

The crystal structure of N/Cl-MoS_2_ was identified by XRD. As shown in Figure 2A, the three MoS_2_ catalyst materials all correspond well to the hexagonal structure (JCPDS 37-1492) and display similar diffraction peaks. (Han et al., 2018) Compared with the pristine MoS_2_, the BM-MoS_2_ and N/Cl-MoS_2_ presented worse crystallinities with reduced intensity and broadened peaks. The crystallinities of the MoS_2_ samples after ball milling tend to be disordered. (Sun et al., 2022) After introducing the heteroatoms N and Cl, the XRD pattern of N/Cl-MoS_2_ was similar to that of pristine MoS_2_, with no new diffraction peaks present, indicating that no new phases (such as MoN or MoCl_5_) were formed. This result indicated that mechanical ball milling has a shear peeling effect on the material, generating smaller and thinner nanometer sized, layered molybdenum disulfide, consistent with the SEM and HR-TEM results. (Xie et al., 2012) In addition, Raman spectroscopy is an effective tool for characterizing the material structure and its defects. (Mignuzzi et al., 2015) As shown in Figure 2B, for all three MoS_2_ samples, characteristic peaks at approximately 405 cm^−1^ and 378 cm^−1^ were observed, which belong to the out-of-plane A_1g_ and the in-plane E 12g modes, respectively. (Hong et al., 2012; Mignuzzi et al., 2015) Compared with the pristine MoS_2_, the BM-MoS_2_ Raman peaks were slightly shifted to lower wavenumbers, attributed to a sharp reduction in both the size and thinning due to ball milling, indicating a local change in the electronic structure. (Krishnamoorthy et al., 2016) After the introduction of N and Cl, the characteristic diffraction peaks of the N/Cl-MoS_2_ samples were significantly shifted, proving that the introduction of N and Cl heteroatoms with different electronegativities changed the electronic structure of N/Cl-MoS_2_. No new peak for the N/Cl-MoS_2_ appears in the range 300–500 cm^−1^ (the peak signal of N/Cl-MoS_2_ is too weak; magnified ×3), indicating that no MoN or MoCl_5_ were formed, consistent with the XRD and TEM results. The results so far are consistent; it is reasonable to speculate that N and Cl are present in the electrocatalytic material in doped form. On the other hand, compared with non-doped BM-MoS_2_, the intensities of the E 12g and A_1g_ peaks for N/Cl-MoS_2_ were weaker. This indicates that the sample was more disordered, due to adjustment and optimization of the electronic structure of MoS_2_ upon introduction of electronegative impurity doping atoms that occupy defect sites. (Mignuzzi et al., 2015; Liu et al., 2016) These results provide preliminary evidence that mechanical ball mill shearing and doughty-electronegative halogen doping 1) produced a N/Cl-MoS_2_ product that exhibits a nanoscale size and thickness, 2) exposed a large number of sulfur edge sites, and 3) optimized the electronic properties of molybdenum disulfide materials by introducing nitrogen and chlorine doping atoms at defect sites.

**FIGURE 3:**
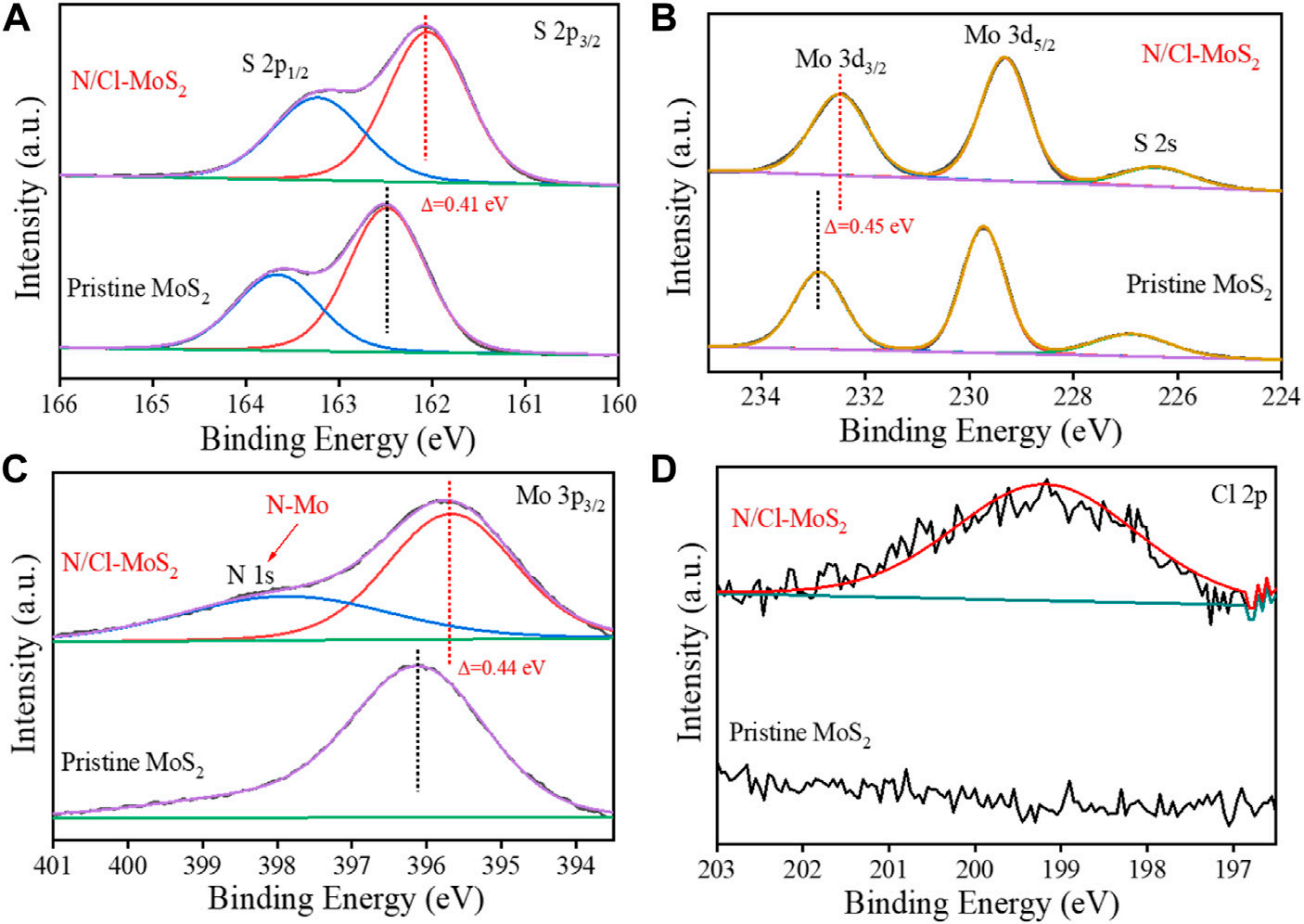
XPS spectra of the pristine MoS_2_ and N/Cl-MoS_2_. **(A)** XPS spectra of S 2p in MoS_2_ samples. **(B)** XPS spectra of Mo 3 d in MoS_2_ samples. **(C)** XPS spectra of Mo 3p and N 1s in MoS_2_ samples. **(D)** XPS spectra of Cl 2p in the MoS_2_ sample.

To investigate the defect-induced effect of doughty-electronegative N and Cl atoms on the electronic structure of the electrocatalyst, we performed X-ray photoelectron spectroscopy (XPS) analysis on the MoS_2_ samples. High-resolution XPS analysis of N/Cl-MoS_2_ and pristine MoS_2_ was performed, and the results are shown in Figure 3. The characteristic S 2p_1/2_ and S 2p_3/2_ peaks were identified at 163.6 eV and 162.5 eV, respectively, as shown in Figure 3A. Compared with pristine MoS_2_, the S 2p_3/2_ peak for N/Cl-MoS_2_ tended toward lower binding energies and was shifted by 0.41 eV (Zhang et al., 2021b) Similarly, the Mo 3d_3/2_ (232.3 eV in Figure 3B) and Mo 3p_3/2_ peaks (396.1 eV in Figure 3C) for N/Cl-MoS_2_ were shifted to lower binding energies by 0.45 eV and 0.44 eV, respectively. The lower binding energies of the Mo 3d_3/2_ and S 2p peaks for heteroatom-induced defective MoS_2_ are attributed to the halogen heteroatom doping process, since halogen heteroatoms exhibit strong electronegativities that shifts the Fermi level of MoS_2_ toward the conduction band edge, consistent with previous reports. (Chen et al., 2013; Zhang et al., 2021b) These results further prove the successful doping of N and Cl and their effect on the electronic structure of MoS_2_. In addition, as shown in Figures 3A,C characteristic N 1s peak was observed in N/Cl-MoS_2_, which indicates the formation of the N-Mo bond. (Xiao et al., 2017) Moreover, a characteristic Cl 2p peak appears at 199.2 eV as shown in Figure 3D, indicating that Cl was successfully doped into MoS_2_. (Zheng et al., 2014) These XPS results show that doughty-electronegative N and Cl atoms were successfully introduced into the defective MoS_2_, and that the electronic structure of MoS_2_ was modulated, providing a prediction for superior HER performance.

**FIGURE 4:**
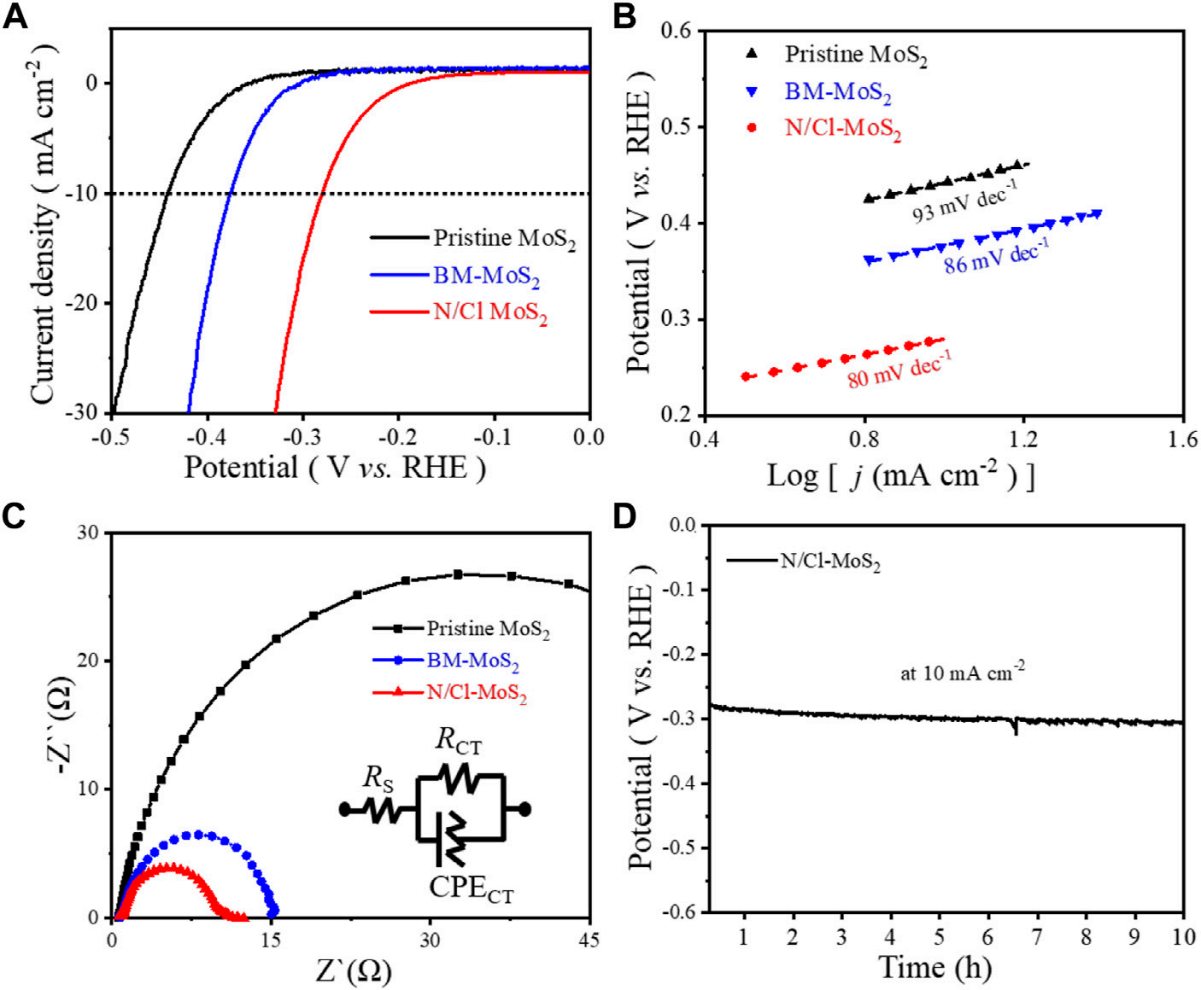
**(A)** LSV polarization curves for pristine MoS_2_, BM-MoS_2_, and N/Cl-MoS_2_. **(B)** Tafel plots for pristine MoS_2_, BM-MoS_2_, and N/Cl-MoS_2_. **(C)** Nyquist plots for pristine MoS_2_, BM-MoS_2_, and N/Cl-MoS_2_. **(D)** Stability measurements for N/Cl-MoS_2_.

Through combined analysis of SEM, HR-TEM, XRD, Raman spectroscopy, and XPS, the unique defect-induced electronic structure caused by doughty-electronegative N and Cl atoms in disordered crystalline MoS_2_ has been determined. The N/Cl-MoS_2_ sample should present the best intrinsic conductivity compared with pristine MoS_2_ and BM-MoS_2_; this should also benefit the catalytic activity. Therefore, we expect the novel N/Cl-MoS_2_ to exhibit superior HER performance. To estimate the HER performances of these MoS_2_ samples (as shown in Figure 4), we obtained linear sweep voltammetry (LSV) polarization curves for the pristine MoS_2_, BM-MoS_2_, and N/Cl-MoS_2_ by using a typical three-electrode system. LSV curves were obtained with *iR*-compensation (95%) at 5 mV s^−1^ in a 1.0 mol L^−1^ KOH solution, as shown in Figure 4A and Supplementary Figure S1. We tested the HER performances of ball milled MoS_2_ samples for different ratios of NH_4_Cl as a N and Cl atom source, as shown in Supplementary Figure S1. Initially, the activity increased as the ratio of NH_4_Cl increased. The best ratio of NH_4_Cl to MoS_2_ was found to be 5:1. We also investigated either a single Cl source or a single N source as a dopant at a ratio of 5:1. The electrochemical results showed that the HER catalytic activity of defect-induced MoS_2_ using double halogen atoms is superior to using a single dopant. As shown in Figure 4A, the LSV curves of the pristine MoS_2_ and BM-MoS_2_ exhibit poor HER performance with inferior overpotentials (approximately 310 mV and 280 mV, respectively) as well as low cathodic current densities. Pristine MoS_2_ required an overpotential of 442 mV and BM-MoS_2_ 372 mV to reach 10 mA cm^−2^, however, the N/Cl-MoS_2_ only required 280 mV; the N/Cl-MoS_2_ exhibits excellent HER activity. These preliminary results showed that, compared with pristine MoS_2_ and BM-MoS_2_, after introducing Cl and N heteroatoms, N/Cl-MoS_2_ exhibited superior activity for the HER. The results also showed that bulk pristine MoS_2_ was smaller and thinner after mechanical ball milling shear stripping, thus exposing more sulfur sites to enhance the catalytic performance of the HER reaction. More importantly, the NH_4_Cl source was broken into electronic N and Cl atoms during ball milling, which doped the molybdenum disulfide to obtain N/Cl-MoS_2_, exhibiting a higher HER performance than undoped MoS_2_. Therefore, the electronic structure of molybdenum disulfide was regulated and optimized by the introduction of N and Cl defect doping atoms, enhancing the intrinsic conductivity and HER catalytic activity. Furthermore, Supplementary Table S2 summarizes the HER catalytic activity of various Mo-based electrocatalysts, which shows that the HER catalytic activity of the N/Cl-MoS_2_ electrocatalyst prepared in this paper is not inferior to other Mo electrocatalysts.

Usually, the Tafel slope curve equation is fitted to the LSV data to explain the kinetics of catalyst materials during the HER. (Junfeng et al., 2013) It is well known that the smaller the Tafel slope, the faster the velocity of the catalytic process, and the more conducive to practical application of catalysts. (Yan et al., 2014) The corresponding Tafel plots of the three MoS_2_ samples are shown in Figure 4B. Compared with the Tafel slopes of pristine MoS_2_ (93 mV dec^−1^) and BM-MoS_2_ (86 mV dec^−1^), the N/Cl-MoS_2_ exhibited a smaller Tafel slope of 80 mV dec^−1^, indicating higher hydrogen evolution activity and a faster electron transfer rate. To investigate the charge transfer ability of the catalysts at the electrolyte/electrode interface, electrochemical impedance spectra (EIS) for the pristine MoS_2_, BM-MoS_2_, and N/Cl-MoS_2_ were obtained. Figure 4C shows the EIS results for the three MoS_2_ catalysts. Furthermore, the interface reactions and electrode kinetics of the MoS_2_ samples were clarified using Nyquist plots; they reveal a dramatically decreased charge-transfer resistance for N/Cl-MoS_2_ compared with BM-MoS_2_ and pristine MoS_2_. (Jiang et al., 2021) As shown in Supplementary Table S3, the impedance fitting data for the MoS_2_-based electrocatalyst was summarized. The results showed that N/Cl-MoS_2_ exhibited a lower charge transfer resistance (R_ct_ = 143Ω) and a faster electron transfer rate than those of pristine MoS_2_ (R_ct_ = 997 Ω) and BM-MoS_2_ (R_ct_ = 219 Ω), indicating that N/Cl-MoS_2_ possessed a faster charge transfer capability in the HER. (Wang et al., 2013) In addition, stability is also an important index for catalyst materials. After continuous galvanostatic operation for 10 h (Figure 4D), the attenuation degree of the electrocatalytic current density is almost negligible for N/Cl-MoS_2_, indicating that the long-term stability is relatively reliable. In other words, the introduction of electronegative heteroatoms in defective MoS_2_ significantly regulates the material’s crystal structure and electron characteristics upon co-doping with N and Cl atoms, improving the conductivity and catalytic activity.

The electrochemical surface areas (ECSAs) were calculated by cyclic voltammetry (CV) and are shown in Supplementary Figure S2. The double layer capacitance (C_*dl*_) of N/Cl-MoS_2_ was 3.1 mF cm^−2^, which is significantly higher than that of pristine MoS_2_ (0.7 mF cm^−2^), but lower than that of BM-MoS_2_ (7.8 mF cm^−2^). This result is consistent with the SEM, Raman, and XRD characterization. Compared with non-doped BM-MoS_2_, although the number of catalytic sites in N/Cl-MoS_2_ was reduced, the electrochemical data (such as LSV polarization curves) indicated that the HER catalytic activity of N/Cl-MoS_2_ was significantly enhanced. Therefore, a reasonable inference is that the introduced halogen atoms enhanced the intrinsic activity, rather than it relying solely on an increase in the number of active sites. These results prove that N/Cl-MoS_2_ possessed more effective active sites due to the introduction of halogen atoms during ball milling, regulating the electronic structure to enhance the intrinsic HER activity.

The ball milling strategy could also be used to tailor the electronic structure of MoS_2_ with other halogen atoms (such as N/F-MoS_2_, the detailed experimental preparation and characterization results of which are provided in the Supplementary Material S1 and Supplementary Figures S3–S6). We tested the electrochemical activity of N/F-MoS_2_ for the HER. Supplementary Figure S7 shows the LSV curves for N/F-MoS_2_, prepared using different ratios of NH_4_F and MoS_2_. The catalytic HER results suggested that the best ratio of NH_4_F to MoS_2_ was 10:1. Its performance was also superior to F-MoS_2_ under identical conditions. The electrochemical surface area of N/F-MoS_2_ (15.4 mF cm^−2^) was higher than that of pristine MoS_2_ (0.7 mF cm^−2^) and BM-MoS_2_ (7.8 mF cm^−2^), as shown in Supplementary Figure S8. Similar to N/Cl-MoS_2_, N and F doping of MoS_2_ also showed reasonable stability (Supplementary Figure S9). These electrochemical results for N/F-MoS_2_ were similar to the results for N/Cl-MoS_2_ discussed above. Compared with single heteroatom introduction, nitrogen and halogen double heteroatom-induced defective MoS_2_ exhibited superior electrocatalytic HER activity, indicating that the electronic structure of the catalyst was regulated by heteroatoms, whether single or double. However, according to the preliminary electrochemical data, modulation using two different doped heteroatoms was more appropriate. In addition, under identical conditions, the N and Cl doped MoS_2_ electrocatalyst was more active compared to doping with N and F, indicating that the introduced doping heteroatom should not be too electronegative, otherwise inferior results will be obtained. Further research into this will be carried out in future.

## 3 Conclusion

In summary, the doughty-electronegative heteroatoms N and Cl were successfully introduced into the crystal structure of MoS_2_ by utilizing a ball milling strategy. The obtained N/Cl-MoS_2_ electrocatalyst presents a significantly improved HER catalytic activity. Compared with the large micrometer-sized bulk pristine MoS_2_, the size of the N/Cl-MoS_2_ is remarkably reduced to the nanometer scale, and the number of exposed active sites is significantly increased. On the other hand, the electronic structure of N/Cl-MoS_2_ is effectively modulated by ball milling and introducing the doughty-electronegative heteroatom N and Cl; the HER activity is significantly enhanced. Compared with pristine MoS_2_, the overpotential of N/Cl-MoS_2_ is observably reduced from 442 mV to 280 mV at 10 mA cm^−2^. This work demonstrates that defect introduction using a doughty-electronegative doping heteroatom could significantly modulate the electronic structure of MoS_2_, thereby enhancing the HER activity and providing a useful strategy for the design of high-efficiency electrocatalysts.

## Data Availability

The original contributions presented in the study are included in the article/Supplementary Material, further inquiries can be directed to the corresponding authors.
