# Supplementary material for: Doughty-electronegative heteroatom-induced defective MoS2 for the hydrogen evolution reaction
Source: Front Chem. 2022 Nov 23;10:1064752. doi: 10.3389/fchem.2022.1064752 (PMC9727101; doi:10.3389/fchem.2022.1064752)
Supplement: Supplementary file 1 [file DataSheet1.pdf]

Supplementary Material for

Doughty-electronegative Heteroatom-induced Defective MoS_2 for Hydrogen Evolution Reaction

1 Experimental Details

1.1 Materials

Commercial MoS_2 (99.5%, 2 um), Ammonium chloride (NH_4Cl, 98.5%) and Ammonium fluoride (NH_4F, 98.5%) were purchased from Guangzhou Chemical Reagent Factory. Nafion solution (5wt% solution, DuPont) was obtained from Sigma Aldrich. The rest of the reagents were provided by Sinopharm Chemical Reagent Co. Ltd. at analytical grade. All chemicals were used without further purification.

1.2 Synthetic Process of MoS_2 samples

In a typical experiment, N/Cl-MoS_2 samples were prepared by a mixed ball milling strategy. 10 g mixture of Pristine MoS_2 and NH_4Cl was mixed with a specific molar ratio (1-1, 5-1, and 10-1) and then placed into stainless steel tank containing 200 stainless steel grinding balls (5 mm in diameter) in the planetary ball-mill machine (MSK-SFM-1, HE FEI KE JING MATERIALS TECHNOLOGY CO., LTD). The tank was sealed followed by fixing it in the planetary ball-mill machine. The mass ratio of mixed material to balls is 40-1, and 1mL of anhydrous ethanol was added as an anti-adhesive. The mixture was ball milled at 450 rpm for 24 h. After the ball milling, the as-prepared product was washed with dilute hydrochloric acid (1 M) three times, then washed with deionized water to remove possible impurities. Further, the solid powder obtained was placed in 400 °C and argon atmosphere for annealing and impurity removal for 2 h. The product was collected, cleaned, and dried for subsequent use. Using a similar strategy, BM-MoS_2 was obtained without a doping source. N/F-MoS_2 was obtained with NH_4F to replace NH_4Cl as a doping source. In the same way, the N-MoS_2, Cl-MoS_2, and F-MoS_2 were obtained with urea, lithium chloride, and lithium fluoride as doping sources, respectively.

1.3 Characterization

The morphology and chemical composition of the samples were investigated by field emission scanning electron microscopy (FE-SEM, S-4800, Hitachi) and energy dispersive X-ray spectroscopy (EDS, QUANTAX 100, Bruker). The structure of the samples was analyzed by high-resolution transmission electron microscopy (HR-TEM, JM -1200EX, JEOL). The crystal structures of the samples were characterized by X-ray diffraction with a Cu Kα source (XRD, D8, Bruker). Raman spectra of the samples were obtained by Renishaw inVia micro-Raman spectrometer with an excitation wavelength of 514 nm. X-ray photoelectron spectroscopy (XPS) analysis was performed using Thermo Scientific Escalab 250Xi and monochrome Al Kα source.

1.4 Electrochemical Measurements

All electrochemical tests were carried out in an electrochemical workstation (CHI 660E, CH Instrument) with a three-electrode system. A glassy carbon electrode was used as a working electrode, a saturated calomel electrode as a reference electrode, and a graphite rod as the counter electrode. The 4 mg electrocatalyst was accurately weighed and then dispersed with 10 uL of Nafion solution in 1 mL ethanol-aqueous solution at a volume ratio of 1:1, and the uniform slurry was obtained by continuous ultrasound for 1 h. Then measure and apply 5μL slurry evenly to the glassy carbon electrode with a diameter of 3 mm, and let it dry naturally for later use. The electrolyte was 1M KOH solution, and the linear sweep voltammetry (LSV) of sample HER was recorded after 5 mV s^{-1} scanning 10 times, and *i*R compensation was 95%. The stability of the N/Cl-MoS_2 electrode was tested by the galvanostatic method. The electrochemical active area (ECSAs) of the samples was measured by cyclic voltammetry (CV) at different sweep rates (10, 20, 30, 40, and 50 mV s^{-1}). The solution compensation resistance of all samples was measured by electrochemical impedance spectroscopy (EIS) at a frequency range of 0.01 Hz to 100000 Hz, and the acquisition voltage was set to - 280 mV *vs.* RHE.

2 Supplementary Figures and Tables

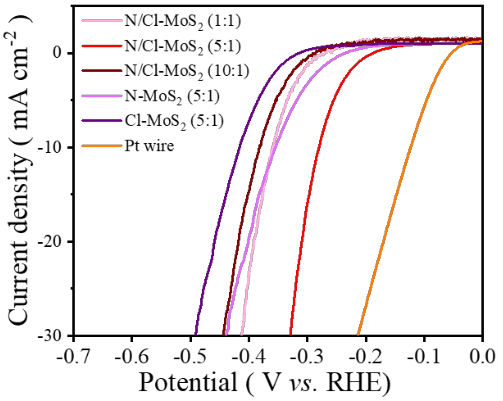

Supplementary Figure S1. LSV curves of different MoS_2 samples and Pt wire.

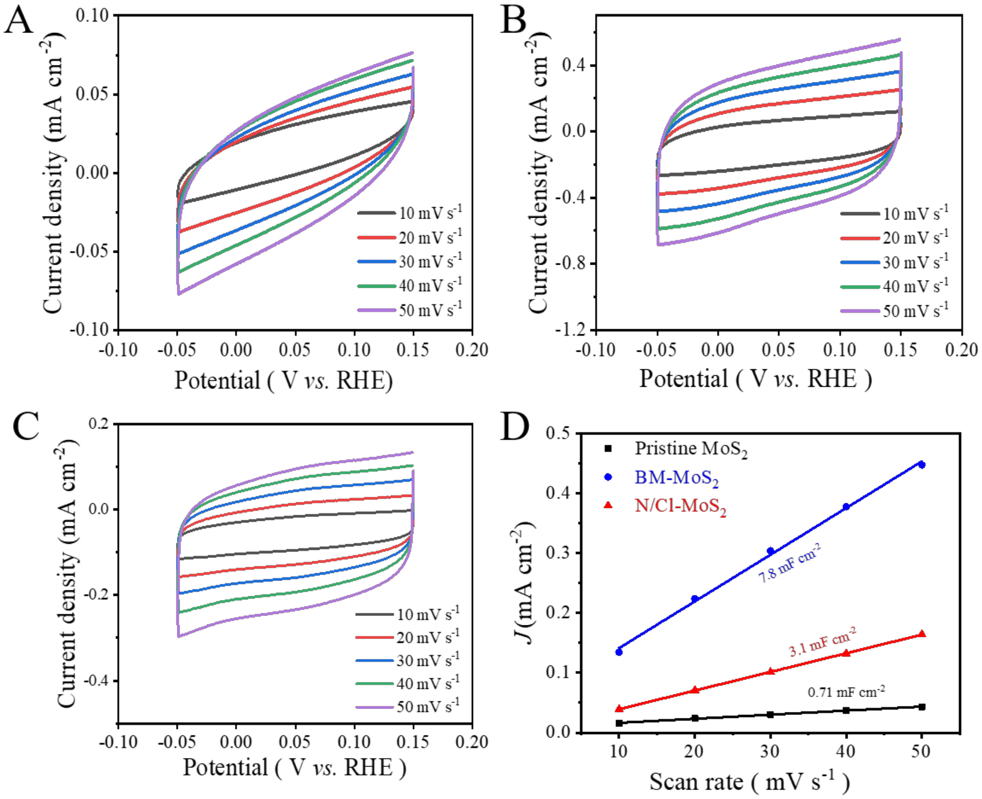

Supplementary Figure S2. Electrochemical surface area (ECSA) tests of different MoS_2 samples. Cyclic voltammogram curves of (A) Pristine MoS_2, (B) BM-MoS_2, and (C) N/Cl-MoS_2 were measured in the non-faradaic potential of -0.05 – 0.15 V *vs.* RHE. (D) ECSA is determined by the capacitive currents at 0.05 V *vs.* RHE.

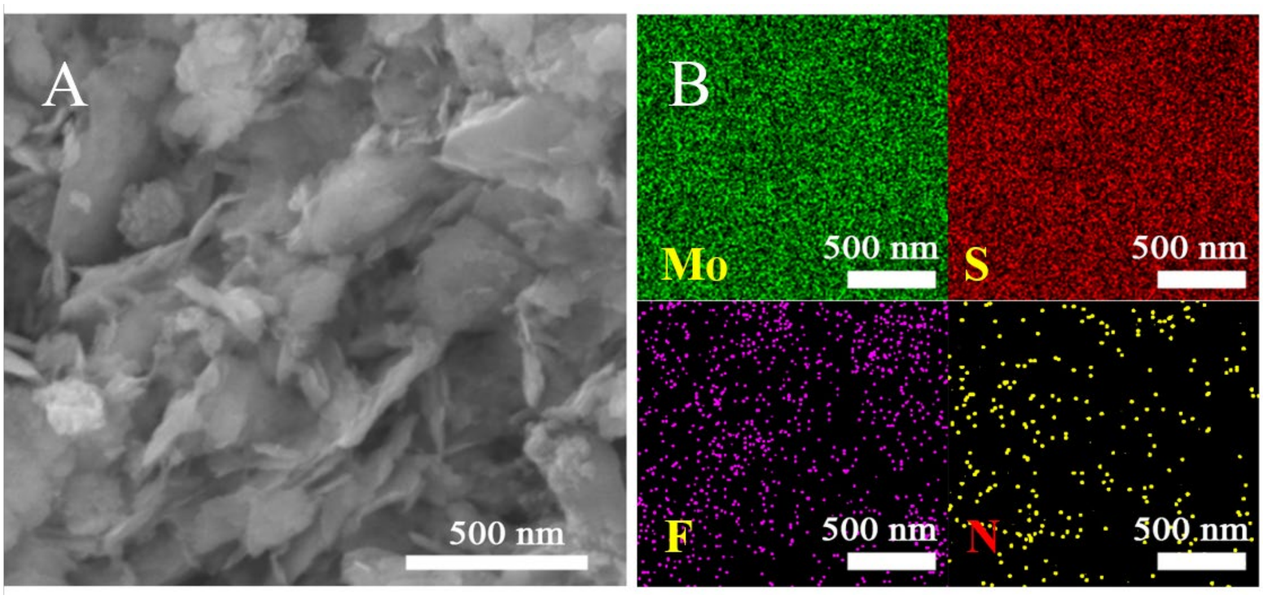

Supplementary Figure S3. (A) SEM images of N/F-MoS_2. (B) EDS element mapping of N/F-MoS_2 from (A). HR-TEM images of (E) Pristine MoS_2, (F) BM-MoS_2, and (G) N/Cl-MoS_2.

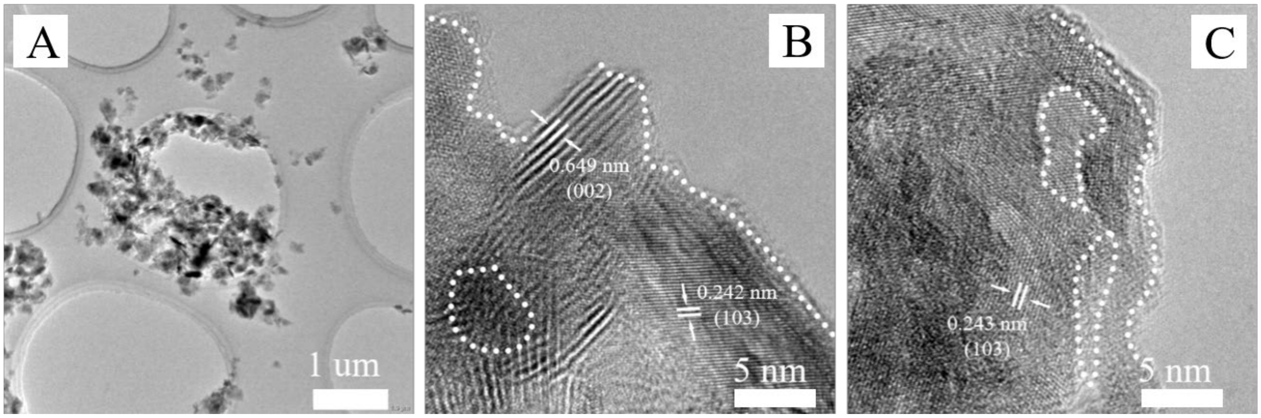

Supplementary Figure S4. (A) TEM images of N/F-MoS_2. HR-TEM images (B) and (C) of N/F-MoS_2 from (A) at different locations.

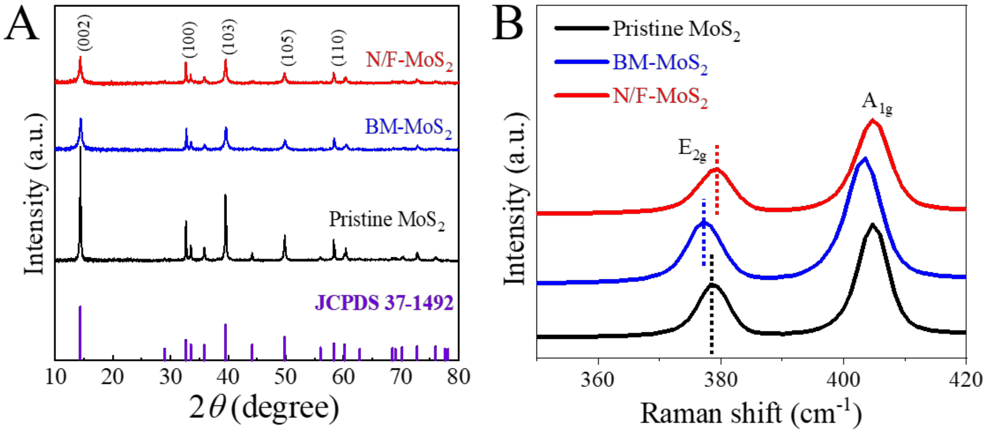

Supplementary Figure S5. (A) XRD patterns of Pristine MoS_2, BM-MoS_2, and N/F-MoS_2. (B) Raman of Pristine MoS_2, BM-MoS_2 and N/F-MoS_2.

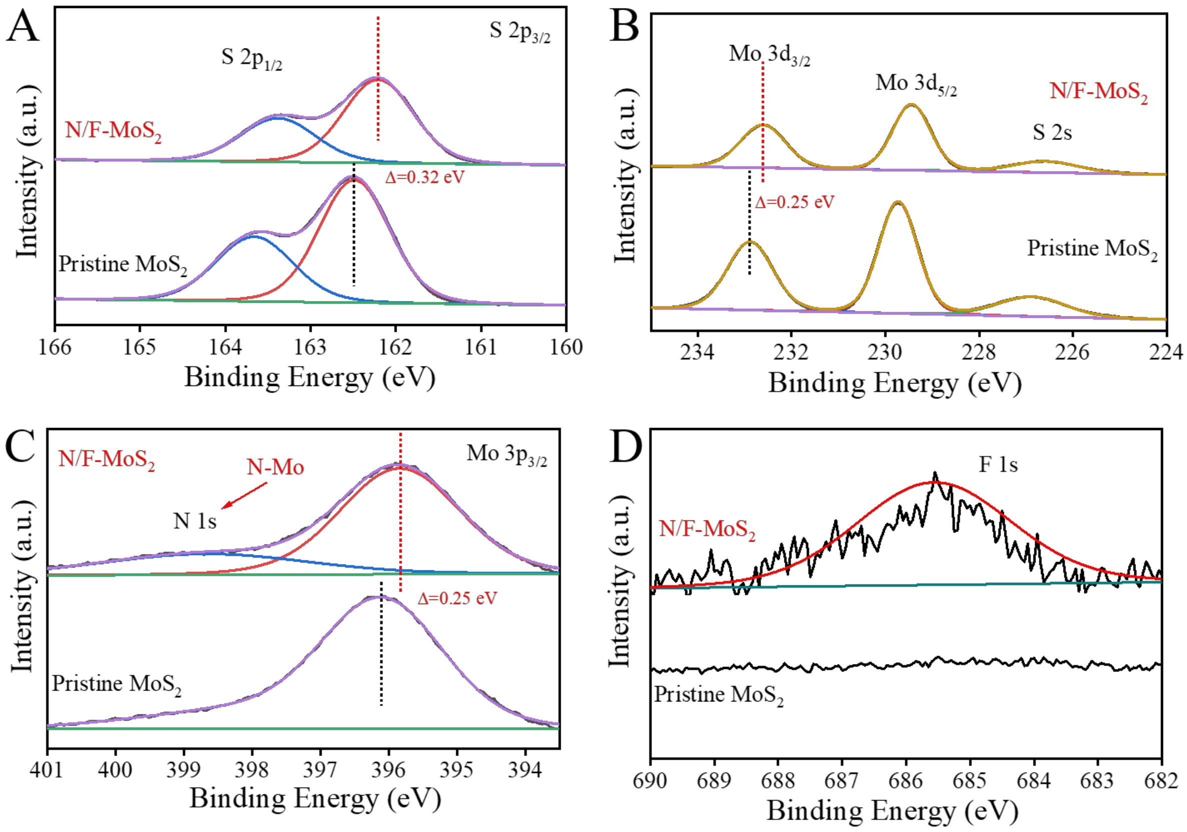

Supplementary Figure S6. Comparative analysis of XPS spectra data of the Pristine MoS_2 and N/F-MoS_2. (A) XPS spectra of S 2p of MoS_2 samples; (B) XPS spectra of Mo 3d of MoS_2 samples. (C) XPS spectra of Mo 3p and N 1s of MoS_2 samples; (D) XPS spectra of F 1s of MoS_2 samples.

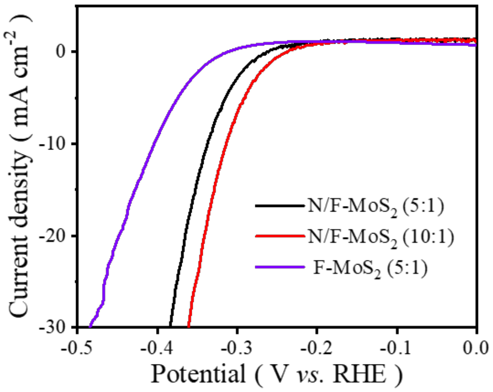

Supplementary Figure S7. LSV curves of MoS_2 samples with different fluorine dopants.

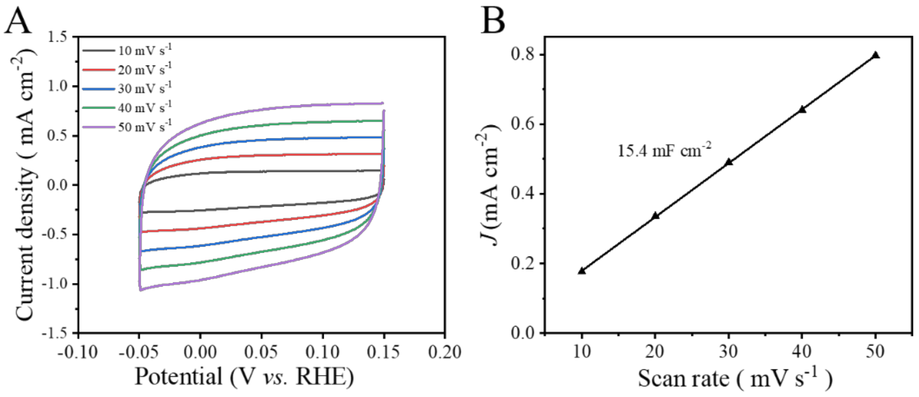

Supplementary Figure S8. Electrochemical surface area (ECSA) tests of N/F-MoS_2. Cyclic voltammogram curves of (A) N/F-MoS_2 measured in the non-faradaic potential of -0.05 – 0.15 V *vs.* RHE. (B) ECSA is determined by the capacitive currents at 0.05 V *vs.* RHE.

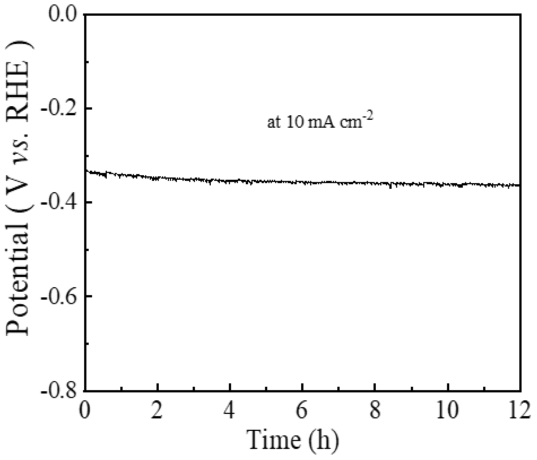

Supplementary Figure S9. Durability measurements of N/F-MoS_2.

Supplementary Table S1. Elemental composition of N/Cl-MoS_2 calculated from EDS.

**Atomic %**	N/Cl-MoS_2
Mo	35.56
S	54.40
N	6.04
Cl	4.00

Supplementary Table S2. MoS_2-based hydrogen evolution electrocatalyst performance.

**Catalyst**	**Overpotential (mV)** in 10 mA cm^{-2}	**reference**
O, P-MoS_2	277	Small, 2020, 16, 1905738
V-MoS_2	320	Nature Communication, 2017, 8, 15113
Li-MoS_2	200	ACS Nano, 2014, 5, 4940
$CoSe_2$/$MoSe_2$	218	Chem Eur J., 2018, 24 (43): 11158
Co_3O_4/MoS_2	348	International Journal of Hydrogen Energy, 2021, 46, 22707
MoS_2/CC-NA	302	J. Phys. Chem. C, 2021, 125, 11369
MoSx@MoS_2-NS	285	Electrochimica Acta, 2018, 292, 136
N/Cl-MoS_2	280	This work

Supplementary Table S3. Summary of the impedance fitting data for MoS_2-based electrocatalyst.

**Sample**	R_S (Ω)	R_{CT} (Ω)	CPE_{CT} (mF)
Pristine MoS_2	9.53	997	98.2
BM-MoS_2	11	219	504
N/Cl-MoS_2	11.5	143	235
